# Rising Shallow Groundwater Level May Facilitate Seed Persistence in the Supratidal Wetlands of the Yellow River Delta

**DOI:** 10.3389/fpls.2022.946129

**Published:** 2022-07-06

**Authors:** Lu Feng, Ling Peng, Qian Cui, Hong-Jun Yang, Jin-Zhao Ma, Jing-Tao Liu

**Affiliations:** Shandong Provincial Key Laboratory of Eco-Environmental Science for the Yellow River Delta, Binzhou University, Binzhou, China

**Keywords:** sea level rise, groundwater level, groundwater salinity, seed persistence, supratidal wetlands

## Abstract

The saline groundwater level of many supratidal wetlands is rising, which is expected to continue into the future because of sea level rise by the changing climate. Plant persistence strategies are increasingly important in the face of changing climate. However, the response of seed persistence to increasing groundwater level and salinity conditions is poorly understood despite its importance for the continuous regeneration of plant populations. Here, we determined the initial seed germinability and viability of seven species from supratidal wetlands in the Yellow River Delta and then stored the seeds for 90 days. The storage treatments consisted of two factors: groundwater level (to maintain moist and saturated conditions) and groundwater salinity (0, 10, 20, and 30 g/L). After retrieval from experimental storage, seed persistence was assessed. We verified that the annuals showed greater seed persistence than the perennials in the supratidal wetlands. Overall, seed persistence was greater after storage in saturated conditions than moist conditions. Salinity positively affected seed persistence under moist conditions. Surprisingly, we also found that higher groundwater salinity was associated with faster germination speed after storage. These results indicate that, once dispersed into habitats with high groundwater levels and high groundwater salinity in supratidal wetlands, many species of seeds may not germinate but maintain viability for some amount of time to respond to climate change.

## Introduction

Persistent seeds, as an important component in the soil seed bank, can represent a reserve of genetic potential that accumulates over time (Sallon et al., 2008). Thus, they have been shown to be an important correlate of population persistence (Saatkamp et al., 2009) that play a crucial role in terrestrial ecosystem conservation and restoration (Thompson et al., 1998; Yang et al., 2021).

Coastal vegetation usually exhibits zonation patterns along an environmental gradient caused by the interaction of land and sea (Antunes et al., 2018). Supratidal wetlands lie beyond the reach of tides, and their hydrological regimes are dominated by precipitation and a shallow saline groundwater level in the vertical direction (Han et al., 2015). Sea level rise, which is induced by events such as climate change and anthropogenic activities (e.g., sea reclamation, embanking, and groundwater pumping), has significant effects on the hydrological conditions of shallow groundwater (Befus et al., 2020). Changes in shallow groundwater directly affect soil moisture and salt conditions, which further affects species distributions (Wilson et al., 2015) and community species diversity (Antonellini and Mollema, 2010; Feng et al., 2018) in supratidal wetlands. It is well-known that persistent soil seed banks play a fundamental role in the assembly of the standing vegetation (Greulich et al., 2019). Mature seeds can therefore be dispersed into conditions that vary along such gradients, which can affect their persistence (Long et al., 2015), however, how soil moisture and salinity caused by groundwater level changes influence seed persistence in supratidal wetlands has received little attention.

The ability of mature seeds to remain viable on the parent plant or on/in the soil referred to as seed persistence (Long et al., 2015; Chen et al., 2021). To some extent, seed persistence varies among species and populations, and depends on reproductive strategies (Rago et al., 2020). Usually, there is formation of a substantial and potentially persistent seed bank when annuals and biennials, which often exhibit r-strategy, produce long-lived seeds with multiyear dormancy; this provides the potential to live through periods of unfavorable conditions (Ooi et al., 2009; Ma et al., 2021). In contrast to annuals and biennials, perennials may have relatively little effect on soil seed banks because their reproductive strategy results in low production of seeds or short-lived seeds (Baskin and Baskin, 2014). Thus, seed persistence can be roughly predicted by species’ reproductive strategies.

Knowledge of the main factors that regulate seed persistence in supratidal wetlands is limited. Usually, seed persistence depends on the physical and physiological characteristics of seeds and how they are affected by the biotic and abiotic environment (Long et al., 2015). In supratidal wetlands, soil moisture and salinity induced by groundwater level gradients may be two factors likely to play roles in seed persistence but have received insufficient attention. Generally, soil moisture affects seed persistence by influencing regulation of germination or dormancy (Rubio-Casal et al., 2003; Hu et al., 2018; Garcia et al., 2020). Kaiser and Pirhofer-Walzl (2015) tested the influence of groundwater level on the seed survival rate of eight wet meadow plant species, and they found that more viable seeds survived at lower groundwater levels compared with higher groundwater levels. *Polygonum aviculare* seeds were induced into dormancy by exposure to low moisture conditions (Batlla et al., 2007). Unfortunately, it is difficult to test the effects of groundwater level gradients on persistence of naturally buried seeds in supratidal wetlands because seeds of different species disperse at different times.

High salinity can inhibit seed germination by reducing the water potential of the soil solution to below the threshold water potential for a species (Long et al., 2015), and regulate seed dormancy and germination by triggering significantly lower abundance of seed proteins involved in several biological processes (e.g., primary metabolism, energy, stress response, and stability) (Debez et al., 2018), and enhance reactive oxygen species (ROS) accumulation by activating the transcription of NADPH oxidase genes (Luo et al., 2021). For example, *Calile maritima* is an annual halophyte on Mediterranean coasts which can produce transiently dormant seeds to inhibit germination under high salinity (Debez et al., 2018). Increasing salt decreased seed germination of *Haloxylon stockii*; however, when shifted from salt solution to distilled water, the ungerminated seeds showed high germination recovery (Rasheed et al., 2019). Thus, inhibition of germination allows seeds to become incorporated into the soil, which is the first stage of seed bank formation.

Plants have evolved several unique mechanisms to adapt to environmental changes. Seed dormancy, a nearly ubiquitous feature across all taxa, is a behavior of plants to prevent seeds from germinating under unfavorable environmental conditions (Gremer and Venable, 2014; Nonogaki, 2014), which contributes to adaptive survival under fluctuating ambient environments. Seed dormancy can range from a few weeks or months to decades or centuries (Finch-Savage and Leubner-Metzger, 2006). It is widely assumed that dormancy may be an effective mechanism that facilitates seed persistence in soil (Cao et al., 2014; Collette and Ooi, 2020). For certain species, such as *Arabidopsis thaliana*, water limitation (Auge et al., 2015) and salt stress (Cheng et al., 2016) can suppress seed germination by enhancing dormancy. Past studies found that salinity acted as an environmental filter that could reduce germinable soil seed bank abundance and Shannon diversity (Kottler and Gedan, 2020; Feng et al., 2021); however, seed viability was not tested in these studies. Thus, we cannot confirm whether high salinity prevented seed germination or killed the seeds.

Seed persistence under experimental conditions can be used to represent the potential of a species to form soil seed banks in nature (Saatkamp et al., 2009). This study complements previous descriptive work (Kottler and Gedan, 2020; Feng et al., 2021) by examining seed fate (viability maintenance, germination, or death) after storage by conducting a burial experiment designed to explore how changes of groundwater level and groundwater salinity affect seed persistence. Also, we used the term germinability to indicate seed germination ability, since some viable seeds may not germinate due to dormancy after storage. We observed changes of seed internal ultrastructure before and after the storage treatments to determine if sharp loss of viability during storage was related to internal physiological structure. We hypothesized that: (1) groundwater level and groundwater salinity have varied effects on seed persistence of the tested plant species, and the strength of this effect depends on the seed bank type of the plant species; (2) the negative relationship found between groundwater level and viable seed number (Kaiser and Pirhofer-Walzl, 2015) suggested that we may observe a negative relationship between groundwater level and seed persistence; and (3) high groundwater salinity favors seed persistence in the soil because inhibited germination allows seeds to become incorporated into the soil seed banks.

## Materials and Methods

### Seed Collection

We selected seven of the most frequent and abundant species in supratidal wetlands of the Yellow River Delta. Seeds were obtained from three annual species, *Suaeda salsa*, *Chenopodium album*, and *Lepidium latifolium*, and four perennial species, *Phragmites australis*, *Cynanchum chinense*, *Apocynum venetum*, and *Imperata cylindrica*. We collected fully ripened seeds of the selected species at maturity in 2019. Seeds were harvested from arbitrarily chosen individuals grown 1 m away from each other and thoroughly mixed. After collection, they were stored in paper bags in the dark at room temperature with 45–50% air humidity until the beginning of the burial experiment. Voucher specimens were deposited at Binzhou University under collection numbers 202006001, 202006002, 202010001, 202010002, 202010003, 202010004, and 202010005.

### Experimental Design

The groundwater in the Yellow River Delta is affected by seawater mainly NaCl (average concentration 20 g/L). We used a factorial design with four replicates to test the effects of different groundwater levels and groundwater salinity on seed persistence of seven species. There were two groundwater levels (low groundwater level, in which the vertical distance from the groundwater to the buried seeds was approximately 3 cm to create a moist condition for the buried seeds; and high groundwater level, in which the groundwater submerged the buried seeds to create a saturated condition for the buried seeds) and four groundwater salinity levels (NaCl concentrations: none added, 10, 20, and 30 g/L).

**FIGURE 1:**
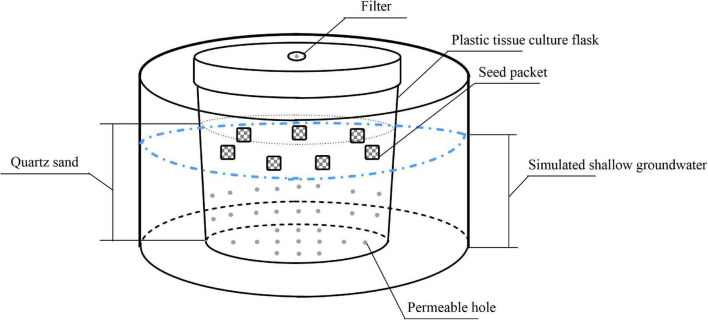
Schematic diagram of the experimental setup showing seed packet layout.

We simulated soil with quartz sand to eliminate the interference of physical and chemical factors. We added 400 g sterile quartz sand (diameter, 2–4 mm) in each 520-mL plastic tissue culture flask and drilled 32 small holes in the body and bottom of the flask. We put each plastic tissue culture flask into matching 1000-mL plastic bowl to simulate different groundwater levels by adding different amounts of water in plastic bowls. In addition, to test the effects of dry storage on seed persistence, seeds were also stored in dry quartz sand without any water storage treatment. The experimental device is shown in Figure 1.

Before storage, some seeds were used to determine the initial germination and viability percentages as a baseline for comparison (mean ± 1 SE across Petri dishes: 85.8 ± 1.6 and 87.5 ± 0.8%, for *P. australis*; 76.7 ± 3.0% and 79.7 ± 2.9% for *S. salsa*; 72.5 ± 3.9% and 76.7 ± 2.4% for *C. chinense*; 64.2 ± 1.6% and 72.5 ± 4.3% for *C. album*; 5.0 ± 1.0% and 47.5 ± 1.6% for *A. venetum*; 0.8 ± 0.8% and 61.7 ± 6.3% for *I. cylindrica*; and 5.0 ± 2.2% and 80.8 ± 1.0% for *L. latifolium*, respectively). We put 50 seeds of each species into small packets made of polyamide fabrics with 1-mm mesh width. After being tagged by colored clips, the seed packets were buried 1.5-cm below the top of the quartz sand in each tissue culture flask and stored in the different treatments for 90 days. To decrease the possible effect of strong light on germination, all devices were kept in the shade (20–30 μmol m^–2^s^–1^).

During storage, the air humidity in the tissue culture flasks was 50–55% for dry storage and 60–65% for low groundwater level storage. After 90 days of storage, the moisture content of the quartz sand with buried seed packets was measured by oven drying. The moisture content of the quartz sand was 0.03% for dry storage, 10.6–11.0% for low groundwater level storage, and 32.9–34.3% for high groundwater level storage. The seed packets were removed from their respective tissue culture flasks, and seed germination and viability percentages were assessed. Although the devices were shaded during the burial experiment, very few seeds germinated. The germinated seeds were considered to have lost persistence. To test germination percentage, we made a culture medium in a 9-cm diameter Petri dish with two layers of filter paper (Feng et al., 2021). We added 2 mL of sterile distilled water to each Petri dish for culturing in a growth chamber (Shanghai Boxun Medical Biological Instrument Co. Ltd., Shanghai, China). The cultivation conditions were: 12: 12 h (day: night), light 60: 0 μmol m^–2^s^–1^, and temperature 25: 20°C. Germination was recorded upon radicle emergence. The number of germinated seeds was counted every third day during the first 2 weeks; thereafter, scoring was carried out once per week until the germination experiment was concluded after 4 weeks. The Petri dishes were placed in a new randomized sequence when the seeds were counted to minimize the effect of possible unequal abiotic conditions in the artificial climate chamber. At the end of the cultivation period, the viability of seeds that did not germinate was checked by opening the seeds and checking the embryos. Seeds with white and firm endosperms were considered viable. Seeds with black/dark brown or mushy endosperms were scored as inviable (Ooi et al., 2004, 2009; Baskin and Baskin, 2014). The seeds that maintained viability but did not germinate were considered dormant (Chantre et al., 2009).

In addition, we selected the seeds with sharp changes in viability percentage to study the changes of seed internal morphological features. The seeds were fixed in 2.5% glutaraldehyde at room temperature for 2 h for subsequent analysis with a scanning electron microscope (SEM). After an extensive rinse with precooled phosphate buffer (pH 7.0), the samples were post-fixed in 1% OsO_4_ overnight at room temperature. The following day, the samples were dehydrated in a graded ethanol series (30, 50, 70, 80, 95, and 100%) and 100% acetone. The samples were then placed overnight in labeled molds that were filled with 100% Spurr’s medium. The samples were vertically sectioned on a ultramicrotome (Leica EM UC7, Leica Microsystems, Wetzlar, Germany) at 90 nm and then examined with a Hitachi SU8010 field-emission SEM (Hitachi SU8010, Hitachi Ltd., Tokyo, Japan).

### Data Analysis

We analyzed the germination percentage (GP), viability percentage (VP), dormancy percentage (DP), and mean germination time (MGT) of seeds after storages. We used the term “germinability” to represent the ability of seeds to germinate after storages under the lab culture conditions. Germinability was inferred based on the germinability index (GI), which represents the rate of germination conservation. We also used the viability index (VI), which represented the rate of viability conservation, to infer seed persistence based on the ability of seeds to maintain viability under storage conditions. The indexes were calculated as follows:


(1) $$\mathrm{GP}\left(\%\right)=\frac{\mathrm{Number}\,\mathrm{of}\,\mathrm{germinated}\,\mathrm{seeds}}{\mathrm{Total}\,\mathrm{number}\,\mathrm{of}\,\mathrm{seeds}}\times 100$$

(2) $$\mathrm{VP}\left(\%\right)=\frac{\mathrm{Number}\,\mathrm{of}\,\mathrm{dyeing}\,\mathrm{seeds}}{\mathrm{Total}\,\mathrm{number}\,\mathrm{of}\,\mathrm{seeds}}\times 100$$

(3) $$\mathrm{DP}\left(\%\right)=\frac{\mathrm{Number}\,\mathrm{of}\,\mathrm{dyeing}\,\mathrm{seeds}-\mathrm{Number}\,\mathrm{of}\,\mathrm{germinated}\,\mathrm{seeds}}{\mathrm{Total}\,\mathrm{number}\,\mathrm{of}\,\mathrm{seeds}}\times 100$$

(4) $$\mathrm{GI}\left(\%\right)=\frac{\mathrm{Final}\,\mathrm{GP}}{\mathrm{Initial}\,\mathrm{GP}}\times 100$$

(5) $$\mathrm{VI}\left(\%\right)=\frac{\mathrm{Final}\,\mathrm{DP}}{\mathrm{Initial}\,\mathrm{DP}}\times 100$$

(6) $$\mathrm{MGT}\,\left(\mathrm{days}\right)=\frac{\sum \left(\mathrm{Nt}\times \mathrm{Dt}\right)}{\sum \text{Nt}}$$

where Nt represents the number of germinated seeds on day t and Dt represents the corresponding number of days.

Statistical analyses were performed using SPSS Statistics version 25.0 (IBM Corp, 2017). Prior to analysis, data were examined for normality, and they were log-transformed as needed. Transformed values were utilized in all subsequent statistical analyses. Untransformed values were presented for all means and standard errors. A two-way Analysis of Variance (ANOVA) was used to examine the effects of groundwater level, groundwater salinity, and their interaction on seed GI and VI (dry treatment was excluded). *T*-tests were used to examine the differences of seed GI and VI between the annual and perennial species, and the effects of groundwater level on GI and VI for each species. One-way ANOVA was used to examine the differences of GI and VI among treatments in each group (W_0_S_0_, W_1_S_0_, and W_2_S_0_; W_1_S_0_, W_1_S_1_, W_1_S_2_, and W_1_S_3_; W_2_S_0_, W_2_S_1_, W_2_S_2_, and W_2_S_3_). Group abbreviations are based on groundwater level and groundwater salinity of each set of conditions. For groundwater level, W_0_ indicates no water, W_1_ indicates low groundwater level, and W_2_ indicates high groundwater level. For groundwater salinity, S_0_ indicates no added salt; S_1_ indicates 10 g/L; S_2_ indicates 20 g/L, and S_3_ indicates 30 g/L. Tukey HSD tests were used for multiple comparisons when the data satisfied the homogeneity of variance test; otherwise, Games–Howell tests were used. Regression analysis curve estimation was used to build optimal models between DP and VI (dry treatment was included), and between DP, GI, VI, and MGT and groundwater salinity. Significance was set to *P <* 0.05.

## Results

### Differences Between Annual and Perennial Species

**FIGURE 2:**
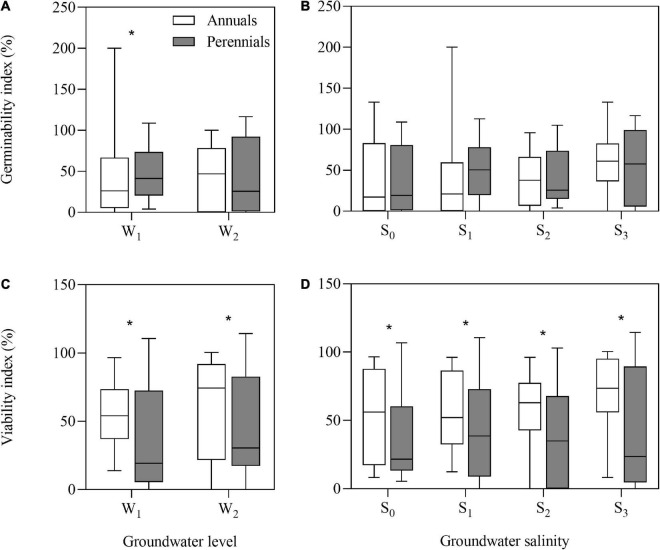
Germinability index **(A,B)** and viability index **(C,D)** of annual and perennial species. W_1_, low groundwater level; W_2_, high groundwater level; S_0_, no added salt; S_1_, salt concentration of 10 g/L; S_2_, salt concentration of 20 g/L; S_3_, salt concentration of 30 g/L. Significant differences between the annual and perennial species are shown by “*.” *P < 0.05.

After storage treatments (including dry storage), seed germinability index (GI) of annuals and perennials had no significant differences, but seed viability index (VI) of annuals (average, 63.0%) was significantly higher than that of perennials (average, 35.0%) (*P* = 0.024). After groundwater level and groundwater salinity treatments, the annual species had greater VI than the perennial species (Figures 2C,D) (all *P* < 0.05). However, GI of the perennial species was significantly higher than that of the annual species after low groundwater level treatment (*P* = 0.032) (Figures 2A,B).

### Effects of Different Groundwater Levels

**FIGURE 3:**
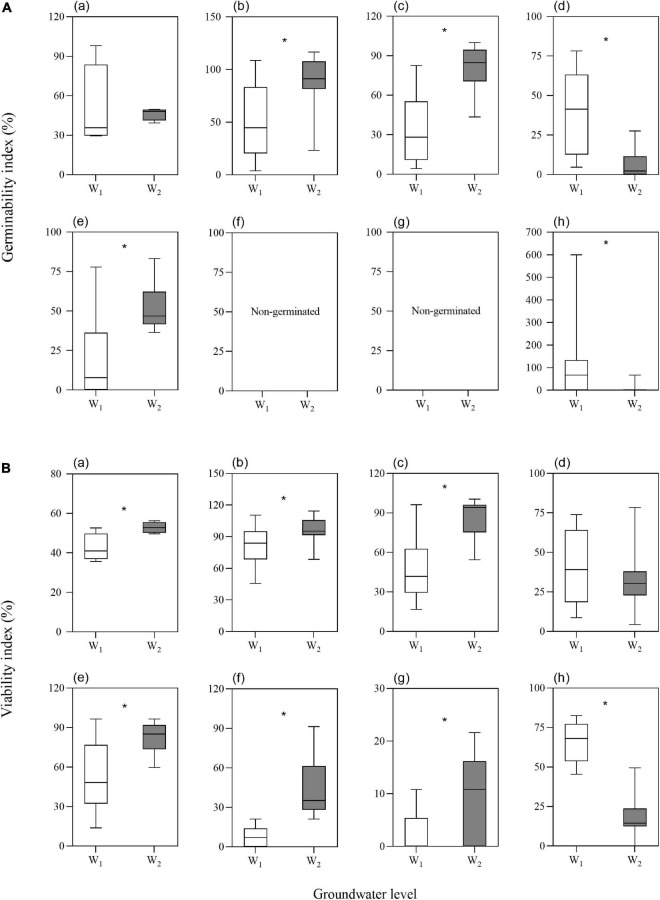
Effects of groundwater level on seed germinability index **(A)** and viability index **(B)** (a. average of the seven species; b. *P. australis*; c. *S. salsa*; d. *C. chinense*; e. *C. album*; f. *A. venetum*; g. *I. cylindrica*; h. *L. latifolium*). Values are the average of GI or VI after storage treatments with the same groundwater level. Significant differences between two groundwater levels are shown by “*” (W_1_, low groundwater level; W_2_, high groundwater level). *P < 0.05.

**FIGURE 4:**
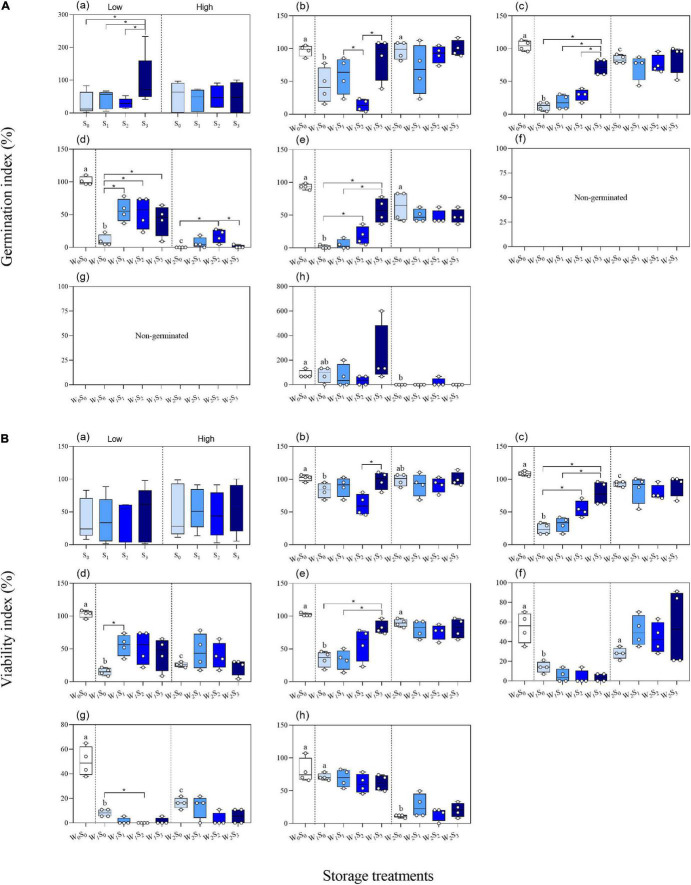
Germinability index **(A)** and viability index **(B)** of seeds (a. average of the seven species; b. *P. australis*; c. *S. salsa*; d. *C. chinense*; e. *C. album*; f. *A. venetum*; g. *I. cylindrica*; h. *L. latifolium*) after different storage treatments. W_0_, no water; W_1_, low groundwater level; W_2_, high groundwater level; S_0_, no added salt; S_1_, salt concentration of 10 g/L; S_2_, salt concentration of 20 g/L; S_3_, salt concentration of 30 g/L. Data are mean ± SE (*n* = 4). Different letters and “*” in each group (W_0_S_0_, W_1_S_0_, and W_2_S_0_; W_1_S_0_, W_1_S_1_, W_1_S_2_, and W_1_S_3_; W_2_S_0_, W_2_S_1_, W_2_S_2_, and W_2_S_3_; W_1_S_1_ and W_2_S_1_; W_1_S_2_, and W_2_S_2_; W_1_S_3_ and W_2_S_3_) indicated significant differences in one-way ANOVAs. Note that the scales of the y-axes are different.

The two-way ANOVA showed that groundwater level storage treatment had significant effects on GI in five of the seven species and VI in six of the seven species (Table 1). Low groundwater level storage generally resulted in decreasing seed GI and VI compared with high groundwater level storage, except for *C. chinense* and *L. latifolium* seeds, which had higher GI and VI after low groundwater level storage (Figures 3A,B). When the effect of groundwater level storage treatment without any salinity (W_0_S_0_, W_1_S_0_, and W_2_S_0_) was analyzed, a clear trend in GI and VI was observed: dry storage (95.9% ± 3.6 and 85.9% ± 4.7%, respectively) > high groundwater level storage (49.3% ± 9.9 and 51.9% ± 7.1%) > low groundwater level storage (30.1% ± 9.3 and 35.7% ± 5.4%) (Figure 4).

**TABLE 1:** Two-way ANOVA results of the effect of groundwater level (GL), groundwater salinity (GS), and interaction between groundwater level and salinity on seed germinability index (GI) and viability index (VI).

	Source	*df*	GI		VI	
			*F*	*P*	*F*	*P*
*P. australis*	GL	1	20.2	**0.000**	7.5	**0.009**
	GS	3	4.1	**0.007**	4.1	**0.012**
	GL × GS	3	3.7	**0.003**	2.1	0.073
*S. salsa*	GL	1	93.8	**0.000**	67.4	**0.000**
	GS	3	9.6	**0.000**	6.5	**0.000**
	GL × GS	3	6.5	**0.000**	7.1	**0.000**
*C. chinense*	GL	1	42.6	**0.000**	1.4	0.479
	GS	3	6.7	**0.000**	4.0	**0.031**
	GL × GS	3	2.6	0.088	0.9	0.273
*C. album*	GL	1	44.2	**0.000**	33.7	**0.000**
	GS	3	5.6	**0.000**	4.5	**0.031**
	GL × GS	3	10.3	**0.000**	6.4	**0.013**
*A. venetum*	GL	1	-	-	42.5	**0.000**
	GS	3	-	-	0.4	0.360
	GL × GS	3	-	-	2.0	0.052
*I. cylindrica*	GL	1	-	-	14.6	**0.012**
	GS	3	-	-	7.3	**0.001**
	GL × GS	3	-	-	1.5	0.626
*L. latifolium*	GL	1	8.4	**0.000**	130.3	**0.000**
	GS	3	1.5	0.477	1.1	0.417
	GL × GS	3	1.8	0.152	1.1	0.499

*Bold values indicate statistically significant effects (P < 0.05).*

### Effects of Varying Salinity

**FIGURE 5:**
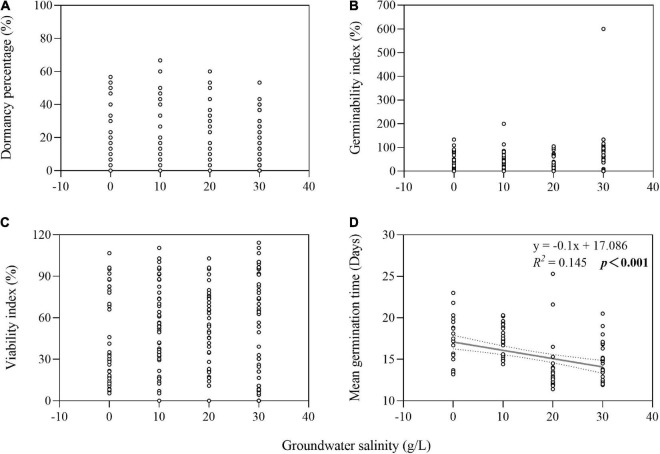
Relationship of groundwater salinity with seed dormancy percentage **(A)**, germinability index **(B)**, viability index **(C)**, and mean germination time **(D)**. The dotted lines indicate the 95% confidence interval of the fit. Note that the x-axes do not start at zero.

The two-way ANOVA showed that groundwater salinity had a significant effect on GI in four of the seven species and on VI in five of the seven species (Table 1). Overall, groundwater salinity had no significant effect on GI and VI when seeds were stored with high groundwater level. However, high salinity generally seemed better for maintenance of germinability and viability under the low groundwater level, except in *I. cylindrica*, whose seeds had better viability when stored in fresh groundwater (0 g/L) than higher salinity groundwater (20 g/L) (*P* = 0.004) (Figure 4). Furthermore, groundwater salinity was negatively correlation with seed MGT (average of seven species after storage treatments including dry storage) (*R*^2^ = 0.145; *P* < 0.001) (Figure 5).

### Interaction Between Groundwater Level and Salinity

**FIGURE 6:**
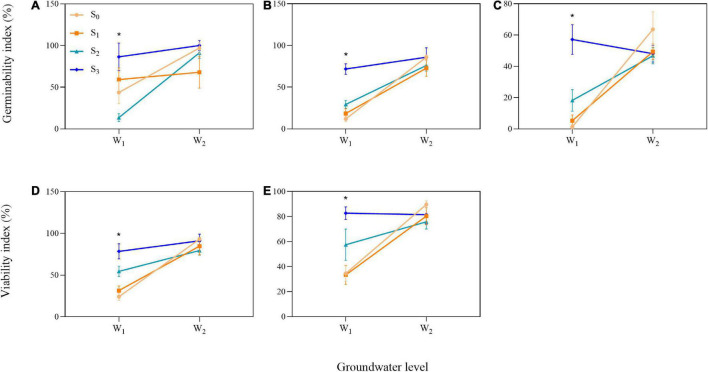
Significant interactions between groundwater level and groundwater salinity on seed germinability index in three species [**(A)** *P. australis*, **(B)** *S. salsa*, and **(C)** *C. album*] and viability index in two species [**(D)** *S. salsa* and **(E)** *C. album*]. W_1_, low groundwater level; W_2_, high groundwater level; S_0_, no added salt; S_1_, salt concentration of 10 g/L; S_2_, salt concentration of 20 g/L; S_3_, salt concentration of 30 g/L. Data are mean ± SE (*n* = 4). Significant differences among different groundwater salinity treatments are shown. *P < 0.05. Note that the scales of the y-axes are different.

We found influence of the interactions between groundwater level and groundwater salinity on seed GI of three species and seed VI of two species (Table 1). When the *P. australis*, *S. salsa*, and *C. album* seeds were stored in the low groundwater level treatments, a positive effect on seed GI was clearly observed for groundwater salinity treatments (Figures 6A–C). The same effects of groundwater salinity were observed on seed VI of *P. australis* and *C. album* (Figures 6D,E). However, the effects disappeared when the seeds were stored in high groundwater level treatments.

### Plausible Relationship Between Seed Dormancy and Persistence

**FIGURE 7:**
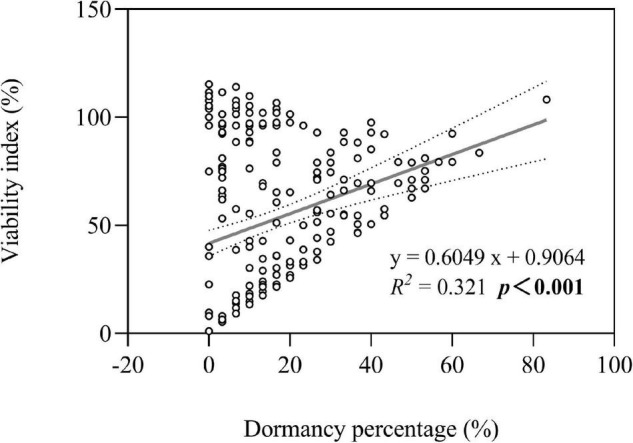
Optimal model for the relationship between seed dormancy percentage and seed viability index. The dotted lines indicate the 95% confidence interval of the fit. Note that the x-axis does not start at zero.

Both seed dormancy percentage (not shown in figures) and seed viability index were greater in three perennial species under high groundwater level storage treatment than under low groundwater level storage treatment (all *P* < 0.001) (Figure 3B). Overall, we found that seed persistence was positively correlated with seed dormancy percentage (average of seven species after storage treatments including dry storage) (*R*^2^ = 0.321; *P* < 0.001) (Figure 7).

### Seed Internal Ultrastructure

**FIGURE 8:**
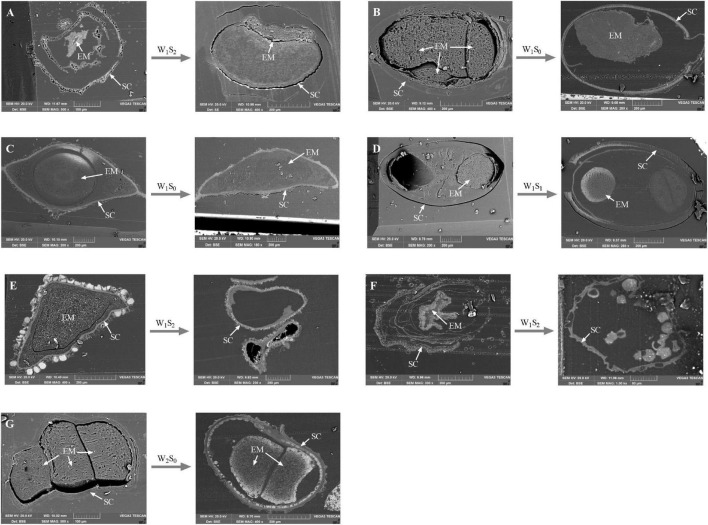
Scanning electron microscopy micrographs of seed vertical sections. The seeds that had sharp change in viability percentage were scanned. **(A)** *P. australis* seeds before and after W_1_S_2_ storage; **(B)** *S. salsa* seeds before and after W_1_S_0_ storage; **(C)** *C. chinense* seeds before and after W_1_S_0_ storage; **(D)** *C. album* seeds before and after W_1_S_1_ storage; **(E)** *A. venetum* seeds before and after W_1_S_2_ storage; **(F)** *I. cylindrica* seeds before and after W_1_S_2_ storage; **(G)** *L. latifolium* seeds before and after W_2_S_0_ storage. EM, seed embryo; SC, Seed coat.

The internal ultrastructural observations made by SEM analysis were mainly carried out on the seeds that had the greatest changes in seed viability after storage treatment. After certain storage treatments (*P. australis* seeds after W_1_S_2_ storage; *S. salsa* seeds after W_1_S_0_ storage; *C. chinense* seeds after W_1_S_0_ storage; *C. album* seeds after W_1_S_1_ storage; *A. venetum* seeds after W_1_S_2_ storage; *I. cylindrica* seeds after W_1_S_2_ storage; *L. latifolium* seeds after W_2_S_0_ storage), we found that the sections of seed embryos were reduced (Figures 8B–D,G) or their shapes were obviously changed (Figures 8A,E,F).

## Discussion

In five of the seven species, we found a clear effect of saturated conditions (high groundwater level storage treatment) maintaining greater seed persistence compared with moist conditions (low groundwater level storage treatment). The lower viability after moist storage was similar to the findings of Diantina et al. (2022), who concluded that seeds of five species experienced significant viability loss after aging under controlled laboratory conditions (storage at 60% relative humidity and room temperature). In our study, the moisture content of quartz sand under moist storage treatment was 10.6–11.0% and relative humidity in the tissue culture flasks was approximately 60–65%, which was similar to aging conditions as mentioned above. Moist conditions may have two effects: first, moist conditions can increase seed moisture content, therefore promote seed metabolism, ROS production and cellular damage (Kibinza et al., 2006; Lee et al., 2019; Renard et al., 2020). Second, moisture potentially affects germination of fungal spores and growth of fungi (parasitic or saprobic) that colonize seeds, which may affect seed viability (Maighal et al., 2016). Moist conditions can stimulate more microbial activity, which causes more seed mortality than saturated conditions (Long et al., 2015; Tatsumi et al., 2022). However, when groundwater salinity increased, the negative effects of moist conditions weakened.

Groundwater salinity had significant effects on seed germination conservation in four species and on viability conservation in five species; however, such effects were mainly found when seeds were stored in low groundwater level treatments. Overall, these results indicated that groundwater salinity change had no significant effects on seed persistence when the soil was saturated, but salinity had a positive effect on seed persistence when the soil was moist. Similar result was found by Gu et al. (2018). In their study, 33 ± 10% relative humidity and high salinity were the optimal storage conditions for *Ruppia sinensis* seeds because these conditions could inhibit seed germination. A possible reason for these results is that high salinity played a role in inhibiting microbial activity (Whittle et al., 2022) under moist conditions to maintain higher seed viability. In addition, it was possible that salt stress triggered an increase in the phenolic compounds and flavonoids level (Tlahig et al., 2021) to protect seeds from microorganisms, and this stress may contribute to antioxidant defense under conditions similar to those in the aging experiment as mentioned above. This conclusion was supported by the interaction between groundwater level and salinity. To some extent, our study provides empirical evidence that soil moisture has a greater influence than groundwater salinity on seed persistence in the supratidal wetlands of the Yellow River Delta.

In addition to these factors, our experiment included a test of groundwater without salts compared to dry storage conditions. Overall, seeds maintained greater germinability and viability in dry storage compared with moist and saturated conditions without salts added. This might be explained by aging being slowed down in desiccated forms of organisms. The possible reasons were that dry condition was conducive to chemical/enzymatic turnover of lipids (Wiebach et al., 2020); metabolism was reduced because of high viscosity and slow diffusion inside cells (Wood and Jenks, 2008), and therefore the production of ROS and cellular damage are inhibited (Renard et al., 2020).

After 3 months of experimental storage, the seed dormancy percentage of three perennial species was greater under high than low groundwater level storage. These results indicated that, once dispersed into habitats with a high groundwater level, many perennial species will enter dormancy even though the habitats were well-suited for germination. This finding highlighted a mechanism that was potentially responsible for the results found in our previous study, in which perennial plants were not observed aboveground in habitats with high groundwater levels (−20 cm) in the supratidal wetlands of the Yellow River Delta (Feng et al., 2021).

There has been some debate about the relationship between seed dormancy and seed persistence in soil. Thompson et al. (2003) found that there was not a close relationship between seed dormancy and persistence. Some ecologists assumed that seed persistence is phylogenetically related to dormancy because persistent seeds are characterized by some kind of dormancy, being either physical, physiological, or a combination thereof (Long et al., 2015; Gioria et al., 2020). Penfield (2017) noted that dormancy experiments should always be accompanied by analysis of environmental conditions. In our burial experiment, the optimal models showed that seed persistence was positively associated with dormancy percentage. These findings supported previous evidence that seed dormancy was an important mechanism that promoted seed persistence in supratidal wetlands. At the population level, seed dormancy enabled soil seed bank formation in response to environmental disturbances. Additionally, at the individual level, seeds maintained or broke dormancy in response to environmental conditions that limited germination to a specific annual time window (Penfield, 2017).

Previously, much work has been conducted on systematics to identify different species by seed characters using SEM (Ahmad et al., 2020), but there has been little reports on the correlation between seed internal ultrastructure and persistence. It was observed that ABA produced by seed embryos contributed to germination arrest upon imbibition and then maintained viability (Chahtane et al., 2017). Our results confirmed the importance of seed embryos in maintaining viability. These unique findings resulted in exploration of the seed viability maintenance mechanism. However, our results were based on a subset of seeds and we did not observe internal microstructure changes for all seeds. Therefore, further research is needed to reveal the quantitative relationship between seed ultrastructure and persistence.

Supratidal wetlands are experiencing particularly rapid change as the shallow groundwater level rises (Befus et al., 2020). Our study indicated that, although the species loss from aboveground vegetation as a result of groundwater rising in supratidal wetlands of the Yellow River Delta, seeds of these species may be buried and remain viable (Basto et al., 2015). Following natural or man-made disturbances, seeds may recover (depending on species) from viable seeds by enhanced germination speed and germinability. Once mature seeds are deposited in supratidal wetlands, the seeds may face three fates. First, in habitats with high groundwater level, a lot of seeds, especially perennials, will not germinate but will maintain higher germinability and viability; this may explain the results of our previous study (Feng et al., 2021). Second, in habitats with low groundwater level and low groundwater salinity, seeds will germinate or maintain lower germinability and viability in the soil. Third, in habitats with low groundwater level but high groundwater salinity, seeds will maintain high viability but may germinate soon after the groundwater salinity decreases (Rasheed et al., 2019) or surface soil salinity decreases caused by precipitation.

## Data Availability Statement

The raw data supporting the conclusions of this article will be made available by the authors, without undue reservation.

## Author Contributions

LF and J-TL designed the experiment with the help of H-JY. LF, LP, QC, and J-ZM collected the seeds in the field and performed the lab experiments. All authors contributed significantly to the writing of the manuscript and approved the submitted version.

## Conflict of Interest

The authors declare that the research was conducted in the absence of any commercial or financial relationships that could be construed as a potential conflict of interest.

## Publisher’s Note

All claims expressed in this article are solely those of the authors and do not necessarily represent those of their affiliated organizations, or those of the publisher, the editors and the reviewers. Any product that may be evaluated in this article, or claim that may be made by its manufacturer, is not guaranteed or endorsed by the publisher.
